# Comparison of Three Non-Invasive Transcranial Electrical Stimulation Methods for Increasing Cortical Excitability

**DOI:** 10.3389/fnhum.2016.00668

**Published:** 2016-12-27

**Authors:** Yasuto Inukai, Kei Saito, Ryoki Sasaki, Shota Tsuiki, Shota Miyaguchi, Sho Kojima, Mitsuhiro Masaki, Naofumi Otsuru, Hideaki Onishi

**Affiliations:** ^1^Department of Physical Therapy, Niigata University of Health and WelfareNiigata, Japan; ^2^Institute for Human Movement and Medical Sciences, Niigata University of Health and WelfareNiigata, Japan

**Keywords:** transcranial direct current stimulation, transcranial random noise stimulation, transcranial alternating current stimulation, motor evoked potential, transcranial magnetic stimulation, cortical excitability

## Abstract

Transcranial direct current stimulation (tDCS) is a representative non-invasive brain stimulation method (NIBS). tDCS increases cortical excitability not only in healthy individuals, but also in stroke patients where it contributes to motor function improvement. Recently, two additional types of transcranial electrical stimulation (tES) methods have been introduced that may also prove beneficial for stimulating cortical excitability; these are transcranial random noise stimulation (tRNS) and transcranial alternating current stimulation (tACS). However, comparison of tDCS with tRNS and tACS, in terms of efficacy in cortical excitability alteration, has not been reported thus far. We compared the efficacy of the three different tES methods for increasing cortical excitability using the same subject population and same current intensity. Fifteen healthy subjects participated in this study. Similar stimulation patterns (1.0 mA and 10 min) were used for the three conditions of stimulation (tDCS, tRNS, and tACS). Cortical excitability was explored via single-pulse TMS elicited motor evoked potentials (MEPs). Compared with pre-measurements, MEPs significantly increased with tDCS, tACS, and tRNS (*p* < 0.05). Compared with sham measurements, significant increases in MEPs were also observed with tRNS and tACS (*p* < 0.05), but not with tDCS. In addition, a significant correlation of the mean stimulation effect was observed between tRNS and tACS (*p* = 0.019, *r* = 0.598). tRNS induced a significant increase in MEP compared with the Pre or Sham at all time points. tRNS resulted in the largest significant increase in MEPs. These findings suggest that tRNS is the most effective tES method and should be considered as part of a treatment plan for improving motor function in stroke patients.

## Introduction

Transcranial direct current stimulation (tDCS) is a non-invasive brain stimulation (NIBS) technique that can alter the excitability of the human cortex (Lefaucheur, 2009). tDCS modulates cortical excitability through the application of weak electrical currents in the form of direct current brain polarization. Depending on the direct current polarity, neuronal firing rates increase or decrease, presumably due to current-induced changes in resting membrane potentials (Liebetanz et al., 2002; Nitsche et al., 2003b). In most settings, anodal tDCS increases, whereas cathodal tDCS decreases motor–cortical excitability (Nitsche and Paulus, 2000, 2001). In a recent study, it was reported that synaptic transmission is likely to be enhanced as a result of increased intracellular Ca^2+^ concentrations in astrocytes (Monai et al., 2016). In addition, anodal tDCS increases cortical excitability not only in healthy individuals but also in stroke patients (Bastani and Jaberzadeh, 2012). Indeed, tDCS is used in the rehabilitation of motor function and contributes to motor function improvement in these subjects (Hummel et al., 2005; Webster et al., 2006; Johansson, 2011; Takeuchi and Izumi, 2012). However, recent studies have indicated significant inter-individual variability in the response to tDCS in healthy individuals (López-Alonso et al., 2014; Wiethoff et al., 2014).

More recently, two additional types of transcranial electric stimulation (tES) methods have been introduced that may also prove beneficial for improving cortical excitability. These are transcranial random noise stimulation (tRNS) and transcranial alternating current stimulation (tACS). Terney et al. (2008) reported that tRNS induces cortical excitability increases lasting 60 min after stimulation (Terney et al., 2008). Moreover, tACS applied with a frequency of 140 Hz, the so-called “ripple frequency”, has been shown to increase excitability in a similar way to both anodal tDCS and tRNS (Moliadze et al., 2010). Interestingly, the after-effects of tRNS and tACS are intensity dependent. Intensity stimulation at 1.0 mA tRNS or tACS leads to excitability after-effects that are comparable to what has been observed with anodal tDCS. However, lower intensity at 0.4 mA tRNS or tACS leads to inhibitory after-effects comparable to those observed with cathodal tDCS (Moliadze et al., 2012). In brief, all of these tES methods (i.e., tDCS, tRNS or tACS) have been reported to increase or decrease cortical excitability.

In previous studies, tRNS has resulted in significantly longer motor evoked potential (MEP) increases than tDCS (Moliadze et al., 2014). However, to date, there is no direct comparison of after-effect of various tES (i.e., tDCS, tRNS and tACS) that enhance cortical excitability using the same current intensity. Finding the most beneficial stimulation method for cortical excitability would be important for determining treatment options for improving the motor function of stroke subjects. The aim of the present study was to compare the efficacy of the three different tES methods for increasing cortical excitability in the same subject population using the same current intensity.

## Materials and Methods

### Subjects

Fifteen healthy subjects (10 males and 5 females; mean age 22.1 ± 3.0 years) participated in this study. Twelve subjects were right-handed and three were left-handed. The Edinburgh Handedness Inventory was used to determine the dominant hand. None of the subjects were taking medications or had a history of physical, neurological or psychiatric disorders. This study was conducted in accordance with the Declaration of Helsinki and approved by the ethics committee of the Niigata University of Health and Welfare. The study was performed at the Institute for Human Movement and Medical Sciences (to which the authors belong). Experiments were canceled immediately if the subject was not in a suitable condition.

### Transcranial Magnetic Stimulation (TMS) and Motor Evoked Potential (MEP) Recording

TMS was applied using a Magstim 200 magnetic nerve stimulator (Magstim Co., Ltd., Whitland, Carmarthenshire, Dyfed, Wales, UK) with a figure-of-eight-shaped coil (diameter, 70 mm). The coil was placed tangentially to the scalp and held at 45° to the midsagittal line for activating the first dorsal interosseous (FDI) muscle. The position and orientation of the coil was monitored using individual magnetic resonance imaging (MRI) with Visor2 TMS Neuronavigation (eemagine Medical Imaging Solutions GmbH, Berlin, Germany). The hot spot of the FDI muscle was recorded and the coil was manually held in place to maintain the position. T1-weighted MRI was obtained using a 1.5-T system before the experiment (Signa HD, GE Healthcare, Milwaukee, WI, USA). The intensity of TMS (defined in terms of the percentage of maximum stimulator output (MSO)) was adjusted to elicit, on average, baseline MEPs of 1 mV peak-to-peak amplitude (Nitsche et al., 2003a; Batsikadze et al., 2013). TMS was performed 12 times at rest, and the maximum and minimum peak-to-peak amplitude values were excluded. The intensity of TMS was kept constant for the post-stimulation assessment.

### Electromyography

Electromyographic activity was recorded via Electromyography (EMG) using surface electrodes placed over the FDI muscle of the right hand. EMG signals were amplified (×100) using an amplifier (A-DL-720-140, 4 Assist, Tokyo, Japan), digitized (sampling rate, 4 kHz) using an A/D converter (Power Lab 8/30, AD Instruments, Colorado Springs, CO, USA), and filtered using a high-pass filter (20 Hz). Data was recorded on a computer and stored for later analysis (LabChart7, AD Instruments).

### Transcranial Electrical Stimulation (tES)

tES was delivered using a DC-STIMULATOR PLUS (Eldith, NeuroConn GmbH, Germany) through a pair of saline-soaked surface sponge electrodes (5 cm × 7 cm, 35 cm^2^). For the three conditions of stimulation (tDCS, tRNS, and tACS), we used similar stimulation patterns (1.0 mA and 10 min), location (FDI hot spot and contralateral orbit), and fade-in/fade-out times of 10 s.

#### Transcranial Direct Current Stimulation (tDCS)

The anode electrode (active) is positioned over the left M1 (FDI hotspot) with the cathode electrode (reference) over the contralateral orbit.

#### Transcranial Random Noise Stimulation (tRNS)

One electrode was fixed above the left M1 (FDI hotspot) and the other electrode was placed over the contralateral orbit. For tRNS, a random level of current was generated for every sample (sampling rate 1280 samples/s). The random numbers were normally distributed and the density function followed a bell-shaped curve. The noise signal contained all frequencies up to half the sampling rate, that is, a maximum of 640 Hz. The signal had no DC offset (Moliadze et al., 2012).

#### Transcranial Alternating Current Stimulation (tACS)

One electrode was fixed above the left M1 (FDI hotspot) and the other electrode was placed over the contralateral orbit. The waveform of the 140 Hz stimulation was sinusoidal (Moliadze et al., 2012).

#### Sham Stimulation (Sham)

The anode electrode (active) is positioned over the left M1 (FDI hotspot) and the cathode electrode (reference) over the contralateral orbit. For sham stimulation, tDCS was turned on for 30 s.

### Experimental Procedures

**Figure 1:**
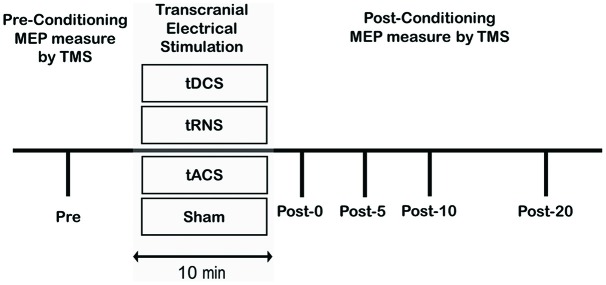
**Experimental procedures.** Subjects participated in the following four sessions: (1) anodal Transcranial direct current stimulation (tDCS); (2) transcranial random noise stimulation (tRNS); (3) transcranial alternating current stimulation (tACS); (4) Sham. For each participant, the maximum stimulator output (MSO) was set to elicit a pre-motor evoked potential (MEP) that averaged 1.0 mV peak-to-peak amplitude. A Pre measure of cortical excitability was obtained prior to the conditioning protocol and then as multiple time-points following conditioning.

The experimental procedure is shown in Figure 1. Subjects participated in four different experimental studies. For all experiments, the order of the stimulation conditions occurred in a counterbalanced fashion, with at least 3 days between two measurements. Stimulus intensities (as a percentage of maximal stimulator output) of TMS were determined at the beginning of each experiment. Following stimulation, 12 single test-pulse MEPs were recorded at 0.2 Hz at approximately 0 min (Post 0), 5 min (Post 5), 10 min (Post 10), and 20 min (Post 20) after stimulation. The electrode was quickly removed after tES. After tES, TMS was performed within 1–2 min. Mean MEP amplitudes, with the maximum and minimum MEP amplitudes excluded, were calculated from the peak-to-peak amplitudes of 10 trials for each of the pre and post stimulation conditions.

### Statistical Analysis

Two-way repeated measures analysis of variance (ANOVA) was used to analyze MEP amplitude. The factors for the ANOVA were four interventions (TYPE OF STIMULATION (tDCS, tACS, tRNS or Sham)) and five time-points (TIME (Pre, Post 0, Post 5, Post 10 and Post 20)). Bonferroni’s methods were used for *post hoc* comparisons.

In addition, the average MEP value of Post 0 to Post 20 was calculated as an after-effect on the stimulation of each condition. One-way repeated-measures ANOVA was used to analyze after-effects, and Bonferroni’s methods were used for *post hoc* comparisons. In addition, Pearson’s product-moment correlation coefficients were calculated for after-effects (tDCS, tRNS and tACS). Statistical analyses were performed using PASW statistics software version 22 (SPSS; IBM, Armonk, NY, USA). The level of significance was set at *p* < 0.05.

## Results

The sample size required for the present study was calculated utilizing G * Power software version 3.1.9.2 (Franz Faul; University of Kiel, Kiel, Germany). The results indicated that 15 subjects would provide a statistical power of 0.80 and an effect size of 0.05 for ANOVA. Also, critical *F* = 1.79 was calculated. The intensity of TMS was not significantly different in tDCS (52.0 ± 1.9%), tRNS (51.8 ± 2.0%), tACS (51.9 ± 2.1%), and Sham (51.9 ± 2.0%). Two-way repeated-measures ANOVA revealed a significant main effect of TYPE OF STIMULATION (*F*_(1.879,26.310)_ = 8.075, *p* = 0.002, partial *η*^2^ = 0.366) and TIME (*F*_(4,56)_ = 14.430, *p* = 0.000, partial *η*^2^ = 0.508). The interaction between TYPE OF STIMULATION and TIME was also significant (*F*_(12,168)_ = 1.888, *p* = 0.039, partial *η*^2^ = 0.119).

**Figure 2:**
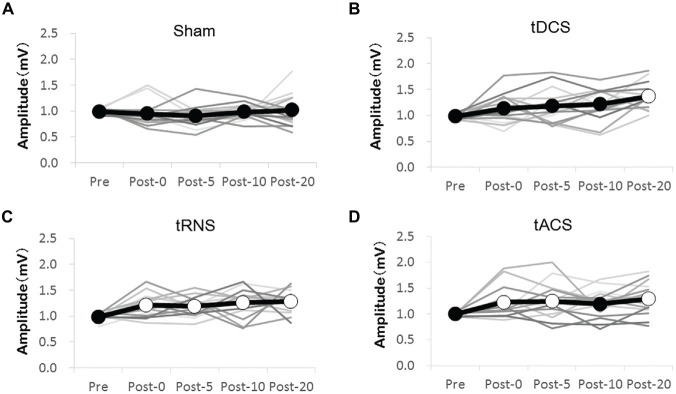
**Effect of the transcranial electrical stimulation (tES) on the MEP amplitudes compared with the Pre measure. (A)** Sham stimulations were without any effect. **(B)** tDCS significantly increased MEP at the Post 20 time-point compared with that at the Pre time-point. **(C)** tRNS significantly increased MEP at the Post 0–Post 20 time-points compared with that at the Pre time-point. **(D)** tACS significantly increased MEP at the Post 0, Post 5, and Post 20 compared with that at the Pre time-point. The gray line shows the amplitude of the MEP for each individual. The black line shows mean amplitudes of the MEP. Open circles indicate significantly increased post-measurements of MEP amplitudes compared with those at the Pre time-point (Bonferroni’s methods, *p* < 0.05).

We compared MEP amplitudes at the single time-points post-stimulation with Pre MEP amplitudes. The changes in MEP for each stimulation condition are shown in Figure 2. According to Bonferroni’s methods, tDCS induced a significant increase in MEP compared with the Pre time-point at time-point Post 20 only (*p* < 0.000). In contrast to the effects of tDCS, tRNS induced a significant increase in MEP amplitude compared with the Pre time-point at all time-points (Post 0–Post 20) (Post 0 (*p* = 0.020), Post 5 (*p* = 0.046), Post 10 (*p* = 0.002), and Post 20 (*p* = 0.001)). tACS induced a significant increase in MEP amplitude compared with the Pre time-point at Post 0 (*p* = 0.044), Post 5 (*p* = 0.025), and Post 20 (*p* = 0.001). In Sham, no significant changes at any of the time-points were observed.

**Figure 3:**
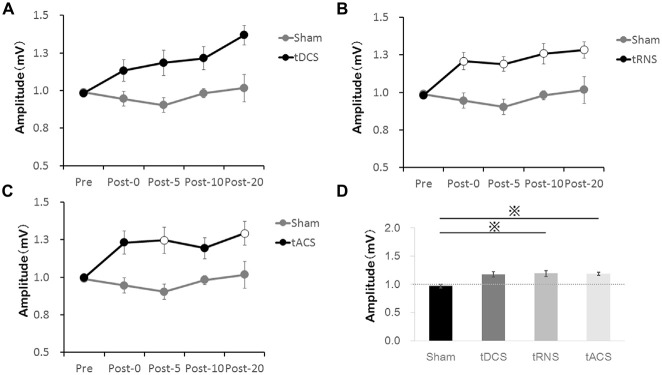
**Effect of the tES method on MEP amplitudes compared with Sham. (A)** tDCS did not significantly increase MEP compared with the Sham. **(B)** tRNS significantly increased MEP at the Post 0–Post 20 time-points compared with the Sham. **(C)** tACS significantly increased MEP at the Post 5 and Post 20 time-points compared with the Sham. Open circles indicate significantly increased post-measurements of MEP amplitudes compared with the Sham (Bonferroni’s methods, *p* < 0.05). **(D)** The bar graphs show the average value of the after-effect of each stimulation condition. According to Bonferroni’s methods, tRNS and tACS had significantly higher values than sham (*p < 0.01). Error bars indicate SE.

A difference in MEP between the stimulation in each time is shown in Figure 3. Comparing all of the stimulation conditions, there were no significant differences in the Pre-condition. Bonferroni’s methods showed significantly higher MEP amplitude at each time Post 0–Post 20 with tRNS than with sham [Post 0 (*p* = 0.035), Post 5 (*p* = 0.011), Post 10 (*p* = 0.046), and Post 20 (*p* = 0.044)]. tACS induced a significant increase of MEP compared with Sham at the time-points Post 5 (*p* = 0.037) and Post-20 (*p* = 0.028). In contrast to the effect of tRNS and tACS, tDCS did not modify the MEP amplitudes significantly compared with sham.

**Figure 4:**
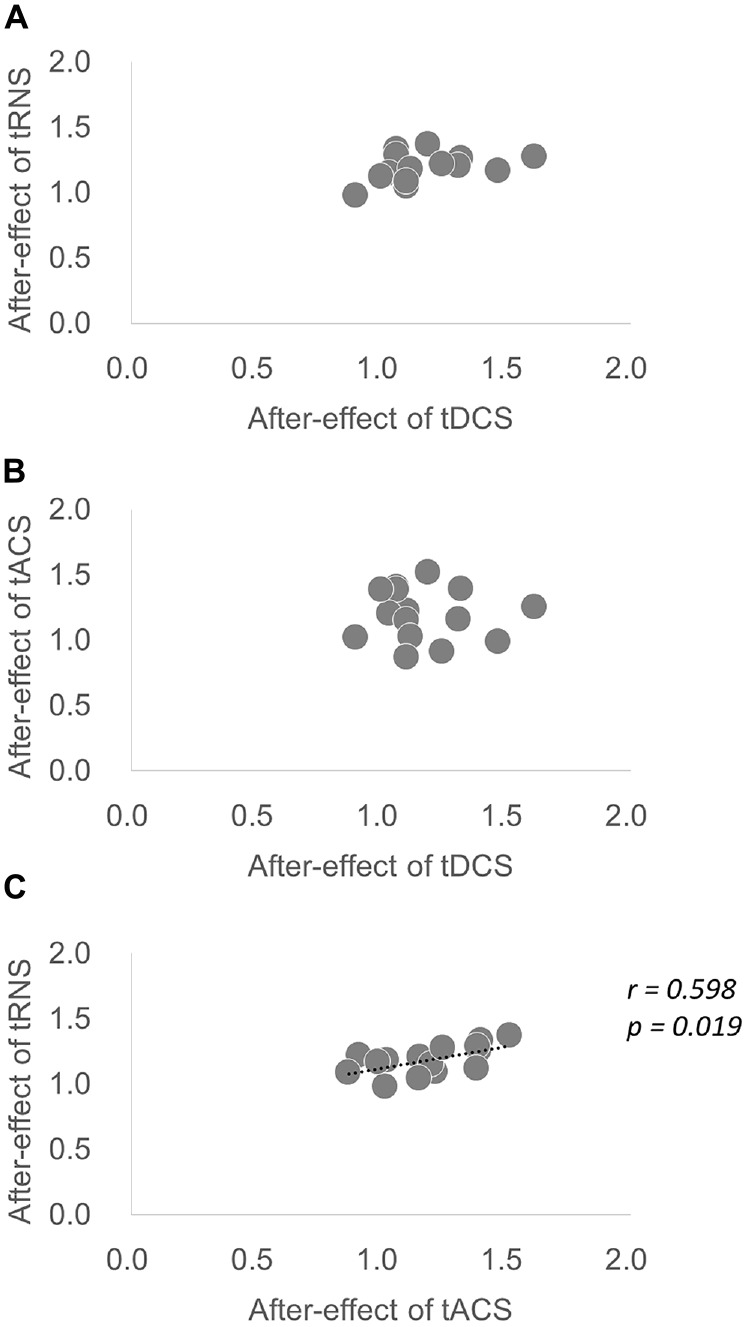
**Scatter diagram of the average after-effect of each stimulation condition. (A)** Scatter diagram of the after-effect of tDCS vs. the after-effect of tRNS. **(B)** Scatter diagram of the after-effect of tDCS vs. the after-effect of tACS. **(C)** Scatter diagram of the after-effect of tACS vs. the after-effect of tRNS. There was a significant correlation between tRNS and tACS.

Regarding the average value of the after-effect, one-way repeated-measures ANOVA was significant [*F*_(1.876,26.268)_ = 8.035, *p* = 0.002, partial *η*^2^ = 0.365]. According to Bonferroni’s methods, tRNS (*p* = 0.001) and tACS (*p* = 0.002) were significantly higher than sham. A scatter diagram of the after-effects of each stimulation condition is shown in Figure 4. There was a significant correlation between tRNS and tACS (*p* = 0.019, *r* = 0.598).

## Discussion

The present study was designed to determine the effects of four different stimulation conditions on cortical excitability in healthy individuals. Our results indicate several important findings. First, there were significant increases in excitability caused by all three conditions in the NIBS technique (tDCS, tACS and tRNS) compared with the Pre-condition, with different times. tRNS increased MEPs at all time points, whereas tDCS and tACS increased MEPs at some of the time points. Second, significant increases in excitability caused by tRNS and tACS were observed compared with that caused by sham, although no significant increases in cortical excitability was observed when tDCS was applied. Compared with sham, the mean stimulation effect was significantly increased by tRNS and tACS; however, no significant increases were observed with tDCS. These findings suggest that tRNS is the most stable enhancement method of cortical excitability stimulation compared with the other methods (tDCS and tACS). In addition, a significant correlation of the mean stimulation effect between tRNS and tACS was observed. tACS is similar, but not identical, to tDCS (Antal and Herrmann, 2016). The regular sinusoidal ups and downs of tACS result in a weak modulation of the membrane voltage. Futher, tRNS is a special form of tACS; during tRNS, a low intensity alternating current is applied where intensity and frequency of the current vary in a randomized manner (Antal and Herrmann, 2016). It is assumed that correlation was found between tRNS and tACS in terms of the mean stimulation effect, because tRNS is a special form of tACS. Also, tRNS has significantly more power than tACS because of the numerous frequencies used. This difference in frequency may be the reason why tRNS increased MEP at more time points than tACS.

The physiological mechanisms of how tRNS generates cortical excitability are not completely understood. One potential effect of tRNS may be associated with the repetitive opening of Na^+^ channels (Schoen and Fromherz, 2008). A recent study demonstrated that the Na^+^ channel blocker carbamazepine showed a tendency towards inhibiting MEP after stimulation (Chaieb et al., 2015). In addition, the effects of tRNS may be based on other mechanisms, such as stochastic resonance (Stacey and Durand, 2000). Stochastic resonance refers to the phenomenon that a signal that is too weak to exceed a threshold is amplified by adding noise. It was suggested that tRNS may increase synchronization of neural firing through the amplification of subthreshold oscillatory activity, which in turn reduces the amount of endogenous noise (Antal and Herrmann, 2016). These two mechanisms may be involved in increased MEP after tRNS.

In previous studies, 140 Hz AC stimulation significantly increased MEP at the Post 0–Post 90, compared with sham (Moliadze et al., 2012). However, MEP significantly increased only at the Post 5 and Post 20 in this study. Different electrode sizes may have affected our results. This previous study used smaller electrodes (4 cm × 4 cm, 16 cm^2^) than we used in this study (5 cm × 7 cm, 35 cm^2^). As a result, the current density becomes smaller. In studies using tDCS, an after-effect change in the current density has been reported (Chhatbar et al., 2016). Thus, it is assumed that the stimulation effect was also different.

Our results indicate that the effect of tDCS increased MEP in only the Post 20, compared with the Pre-condition; however, the comparison to sham was not significant. The after-effects of tRNS and tACS, compared with those of tDCS, were beneficial in this study. The after-effect of tDCS has been reported to be different by stimulation intensity or electrode montage (Nitsche et al., 2008; Vines et al., 2008; Bastani and Jaberzadeh, 2013). In addition, there is increasing recognition of the high variability in the reported effects of tDCS (Li et al., 2015). Our results may also have been affected by inter-individual variability in the response to tDCS. There are many factors that can influence this variability such as the thickness of the skull and sulcal depth (Opitz et al., 2015) as well as the genotype of the brain-derived neurotrophic factor (BDNF; Teo et al., 2014; Puri et al., 2015). Antal et al. (2010) reported that the BDNF polymorphism appears to influence the response to tDCS but has no influence on the response to tRNS (Antal et al., 2010). Collectively, these findings and the results of this study indicate that tRNS is the most beneficial stimulation method.

Currently, tDCS is used for the rehabilitation of subjects with stroke. In previous studies, tDCS was shown to contribute to the improvement of motor function in stroke patients (Hummel et al., 2005; Webster et al., 2006; Johansson, 2011; Takeuchi and Izumi, 2012). However, there have also been negative reports with tDCS (Elsner et al., 2016). Factors such as lesion patterns, severity of paresis and time-course post stroke are very important in the clinical application of tDCS. tDCS appears effective in patients that have had chronic or mild-moderate strokes as opposed to those that have had acute, subacute or moderate-severe strokes (Lindenberg et al., 2010; Flöel, 2014; Marquez et al., 2015; Chhatbar et al., 2016). In this study, tRNS appeared to be the most stable NIBS technique compared with tDCS and tACS. Previous research in healthy subjects, in which tRNS was applied to the visual areas of brain, indicated an improvement in behavioral performance in comparison to tDCS (Fertonani et al., 2011). In addition, the transient suppressive effect on tinnitus loudness and tinnitus-related distress induced by tRNS was larger than that induced by tDCS and tACS (Vanneste et al., 2013). In general, anodal tDCS, which enhances cortical excitability, is used for the purpose of improving motor function of stroke patients. However, in this study, tRNS showed significant cortical excitability increase at many time points compared with tDCS. tRNS enhances cortical excitability more stably than tDCS; therefore, it may improve the motor function of stroke patients more steadily. Further study of its use for improving motor function in stroke patients is needed.

One limitation of this study is that all the subjects were healthy and young; thus, it remains unclear if similar results would be obtained with stroke subjects or elderly subjects. Further study is needed to determine whether age or disease state would impact these results. In addition, this study measured only MEP amplitude over a short time (until after 20 min). Further study is required to determine not only the MEP amplitude of a short time but also the long term effects. Moreover, the evaluation of motor function and behavior in response to stimulation effect should be conducted.

## Conclusion

In this study, we compared the after-effects of different excitatory transcranial electrical stimulation methods (tDCS, tACS and tRNS) in the same healthy subjects. Our findings indicate that tRNS is the most beneficial stimulation method for increasing cortical excitability.

## Author Contributions

HO conceived the study and designed the experiment. YI conducted the experiments. KS, RS and ST performed the interpretation of data. YI, SM and KS performed the statistical analysis. KS, MM and NO helped in writing and revising the manuscript. HO and YI wrote the manuscript. All authors read and approved the final manuscript.

## Funding

This work was supported by a Grant-in-Aid for Exploratory Research from the Niigata University of Health and Welfare, 2016.

## Conflict of Interest Statement

The authors declare that the research was conducted in the absence of any commercial or financial relationships that could be construed as a potential conflict of interest.
